# Dextrocardia With Situs Inversus in a COVID-19 Patient

**DOI:** 10.7759/cureus.52219

**Published:** 2024-01-13

**Authors:** Anastasia Rigatou, Konstantinos C Christodoulou, Xafnoula Zlatidou, Ioannis Nikolakakis

**Affiliations:** 1 Computed Tomography and MRI Department, Sismanogleio General Hospital, Athens, GRC; 2 Laboratory of Anatomy, School of Medicine, Democritus University of Thrace, Alexandroupolis, GRC; 3 Emergency Medicine, Tzaneio Prefecture General Hospital of Piraeus, Athens, GRC; 4 History of Medicine and Medical Ethics Department, School of Medicine, National and Kapodistrian University of Athens, Athens, GRC

**Keywords:** sars-cov-2, clinical examination, electrocardiography (ecg), miror anatomy, anatomic variation

## Abstract

With an estimated incidence of one in 10,000 to one in 50,000 patients, Situs inversus totalis (SIT) is a rare innate anomaly, portraying a mirror image of the normal anatomy, as the cardiac position and abdominal viscera are completely inverted. Despite the fact that physicians and researchers have been dealing with the SARS-CoV-2 pandemic for three years, there is a lack of published data examining the potential effects of anatomic variations on coronavirus disease 2019 (COVID-19) infection. This study aimed to contribute to this domain by presenting a rare case of a COVID-19 infection coexisting with SIT as one of the very few cases reporting the simultaneous presence of the two pathologies. We sought to present this case of COVID-19 in a quinquagenarian female, in whom SIT was an incidental radiological finding. The reversed anatomy did not seem to affect the clinical progression of the virus. However, due to the lack of scientific evidence, the potential long-term effects, if any, of COVID-19 on SIT cannot be predicted. The recognition of the mirror pattern will offer a personalized treatment plan, reducing the risk of severe complications and management mishaps.

## Introduction

Situs inversus totalis (SIT) is a rare congenital anomaly with an estimated incidence of one in 10,000 to one in 50,000 patients, displaying a mirror image of the normal anatomy as the cardiac position and abdominal viscera are completely inverted [1]. It is inherited by autosomal recessive traits. Gender and racial distribution are the same. In most cases, situs inversus is an isolated, unintentional event that happens to an individual in the family for the first time; nevertheless, it can very rarely recur in multiple members of the same family. Despite the fact that this unique clinical entity has been known since the Hippocratic years, healthcare professionals have little familiarity with it [2].

Since the initial pneumonia cases, which signaled the beginning of the SARS-CoV-2 pandemic, the intricacy of coronavirus disease 2019 (COVID-19) has presented multiple challenges to clinicians, not only in terms of the required high-quality healthcare services but also in terms of research. Despite the fact that physicians and researchers have been dealing with the pandemic for three years, there is a lack of published data examining the potential effects of anatomic variations on COVID-19 infection [3]. There have been reported cases of patients with primary ciliary dyskinesia and COVID-19 infection; however, the coexistence of SIT and COVID-19 infection is still insufficiently studied [4]. Herein, our objective was to present a case of a quinquagenarian female who had a COVID-19 infection coexisting with SIT.

## Case presentation

**Figure 1:**
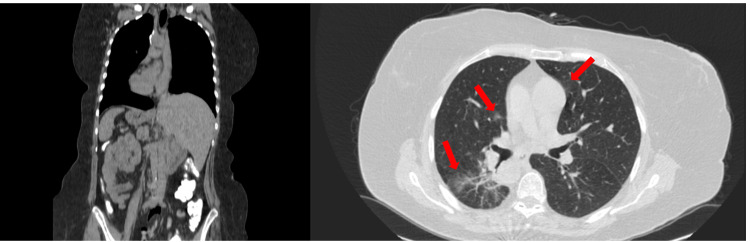
Coronal (left side) and axial views (right side) of computed tomography of the abdomen and thorax. Ground glass infiltrates (arrows) in the right lower and middle pulmonary lobe and the left upper pulmonary lobe; findings suggestive of early stages of COVID-19 infection. COVID-19: coronavirus disease 2019

**Figure 2:**
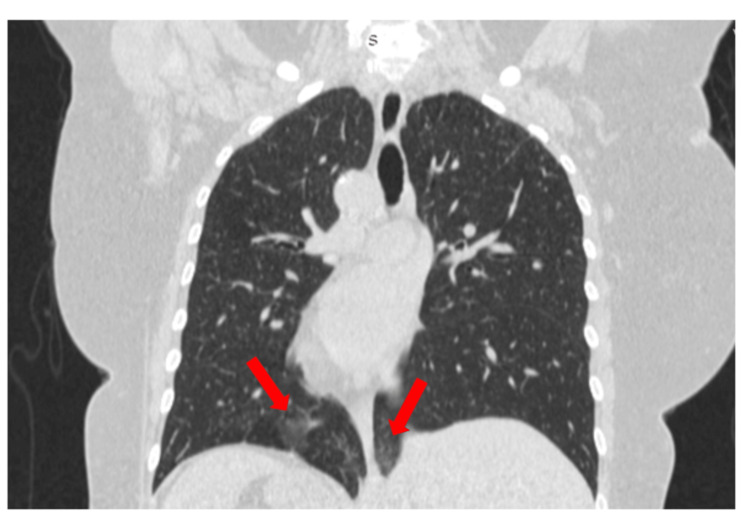
Computed tomography of the thorax, coronal view. Bilateral ground glass infiltrates (arrows) in the lower pulmonary lobes.

A 58-year-old female, self-reporting a right-sided heart with otherwise no prior medical history, was admitted to the Emergency Department of Sismanogleion General Hospital, Athens, Greece, complaining about a nonproductive cough, mild shortness of breath, chest discomfort, dyspnea on exertion and a two-day fever. Physical examination revealed an elevated body temperature (38.5°C), a blood pressure of 145/90 mmHg, a respiratory rate of 17 breaths per minute, and right-sided heart sounds on auscultation, with the point of maximum impulse located to the fifth intercostal space along the midclavicular line. The patient experienced an exacerbation of chest pain during episodes of deep inspiration or coughing. Despite the presumably musculoskeletal nature of the pain, the attending physician set the exclusion of an acute myocardial infarction (MI) as the first priority. An electrocardiogram (ECG) with intentionally reversed precordial and limb leads demonstrated a normal sinus rhythm of 90 beats per minute, along with normal P wave pattern and R wave progression, followed by two negative troponin tests, which ruled out an MI. The patient was tested positive for SARS-CoV-2. A plain X-ray examination was ordered, confirming dextrocardia. Subsequently, the patient underwent a computed tomography scan (CT), which apart from the early but distinct COVID-19 ground glass opacities, demonstrated a situs inversus totalis (SIT) as an incidental radiological finding. The complexity of the case initiated a more thorough diagnostic and imaging investigation; thus, the patient was referred to the pulmonology department (Figures 1, 2).

## Discussion

Situs anomalies are a diverse group of congenital visceral and vascular abnormalities with a wide range of radiographic manifestations [5]. Situs can be classified into three following types: solitus, inversus, and ambiguous [6]. Regarding situs inversus, the following two additional subtypes exist: situs inversus with dextrocardia and situs inversus with levocardia. The terms levocardia and dextrocardia refer to the position of the cardiac apex at the time of delivery, not the position of the cardiac chambers themselves [7].

Dextrocardia with situs inversus has a low incidence of association with congenital heart disease, whereas isolated dextrocardia, in 90-95% of cases, has a higher incidence of association with cardiac anomalies such as atrial or ventricular septal defect and transposition of the great vessels. Situs inversus with dextrocardia and a structurally normal heart is frequently discovered by coincidence in a chest X-ray, which is commonly misinterpreted as normal because the film is reversed when initially examined [8-10]. In the absence of other congenital anomalies, a person with SIT has a normal life expectancy. The presence of cardiac anomalies reduces one’s life expectancy, which is further influenced by the severity of the defect [11].

The abnormal position of the heart, which results in deviant manifestations and clinical findings, has the potential to postpone prompt diagnosis and the critical prompt response in emergency cases [12]. CT is the most effective imaging modality for dextrocardia with situs inversus. Unless dextrocardia is confirmed with a chest X-ray, the ECG, although distinctive, might be mistaken for the significantly more prevalent limb lead reversal, in which the precordial leads exhibit a typical R-wave progression, while in dextrocardia the pattern is reversed [12]. Other findings in the case of a standard lead placement are included but are not limited to the right axis deviation and positive QRS in aVR, inverted P waves in leads I and aVL, and/or a reflection image of the typical R wave pattern in the precordial leads. Thus, the ECG results are revealed when the leads are arranged in a reversed pattern only after dextrocardia has been determined [13]. It seems that despite the various studies reporting a right-sided ECG, a consensus regarding the optimal lead placement is yet to be reached; some authors suggest that all leads should be reversed (as illustrated in our case), while others propose only the reversal of leads V3-V6 [12].

Structural and functional cilia malfunction in visceral lateralization during the early weeks of gestation have been related to situs inversus. Though it is debatable whether SARS-CoV-2 can spread vertically, early gestational fetal infection may have an impact on visceral lateralization; alternatively, visceral lateralization and left-right organizer function may be negatively impacted by SARS-CoV-2-mediated maternal inflammatory responses [14]. Additional investigation is required to confirm that the incidence of these cases was not influenced by genetic abnormalities in primary ciliary dyskinesia-related genes that might not have been found during prenatal genetic screening, as well as to evaluate the possible role of environmental factors [14].

SIT can coexist with other anatomic abnormalities, such as asplenism, multiple spleens, duodenal atresia, ectopic kidney, congenital heart disease, or even vascular and pulmonary malformations, such as Kartagener syndrome [11]. Patients with primary ciliary dyskinesia and COVID-19 have been previously described in the literature [4]. However, to the best of our knowledge, there has been only one study reporting the simultaneous presence of SIT and COVID-19 infection [15]. In the present case, the reversed anatomy did not affect the progression of the virus; therefore, the long-term effects of this correlation cannot be predicted.

## Conclusions

One area that remains underexplored is the potential link between COVID-19 and anatomical variations. This study aimed to contribute to this domain by presenting a rare case of a COVID-19 infection coexisting with SIT, as one of the very few cases reporting the simultaneous presence of the two pathologies. The reversed anatomy did not seem to affect the clinical progression of the virus. However, due to the lack of scientific evidence, the potential long-term effects, if any, of COVID-19 on SIT cannot be predicted. The recognition of the mirror pattern will offer a personalized treatment plan, reducing the risk of severe complications and management mishaps.
